# Dynamics of the Centromeric Histone CENH3 Structure in Rye-Wheat Amphidiploids (Secalotriticum)

**DOI:** 10.1155/2018/2097845

**Published:** 2018-11-26

**Authors:** Yulia A. Lipikhina, Elena V. Evtushenko, Oleg M. Lyusikov, Igor S. Gordei, Ivan A. Gordei, Alexander V. Vershinin

**Affiliations:** ^1^Institute of Molecular and Cellular Biology SB RAS, Novosibirsk 630090, Russia; ^2^Institute of Genetics and Cytology, NAS of Belarus, Minsk 220072, Belarus

## Abstract

The centromeres perform integral control of the cell division process and proper distribution of chromosomes into daughter cells. The correct course of this process is often disrupted in case of remote hybridization, which is a stress factor. The combination of parental genomes of different species in a hybrid cell leads to a “genomic shock” followed by loss of genes, changes in gene expression, deletions, inversions, and translocations of chromosome regions. The created rye-wheat allopolyploid hybrids, which were collectively called secalotriticum, represent a new interesting model for studying the effect of remote hybridization on the centromere and its components. The main feature of an active centromere is the presence of a specific histone H3 modification in the centromeric nucleosomes, which is referred to as CENH3 in plants. In this paper the results of cytogenetic analysis of the secalotriticum hybrid karyotypes and the comparison of the CENH3 N-terminal domain structure of parent and hybrid forms are presented. It is shown that the karyotypes of the created secalotriticum forms are stable balanced hexaploids not containing minichromosomes with deleted arms, in full or in part. A high level of homology between rye and wheat enables to express both parental forms of* CENH3* gene in the hybrid genomes of secalotriticum cultivars. The CENH3 structure in hybrids in each crossing combination has some specific features. The percentage of polymorphisms at several amino acid positions is much higher in one of the secalotriticum hybrids, STr VD, than in parental forms, whereas the other hybrid, STr VM, inherits a high level of amino acid substitutions at the position 25 from the maternal parent.

## 1. Introduction

Many species of the Triticeae tribe are natural allopolyploids and attractive objects for obtaining synthetic hybrids, which are promising material for practical breeding. The most common and studied examples of such hybrids are allopolyploid triticale. The synthetic allohexaploid triticale has a genome structure similar to hexaploid bread wheat except that it has rye as one of its progenitor instead of the D genome donor* Aegilops tauschii*. Triticale is an important model for studying the rapid changes that occur subsequent to polyploidization involving genomic remodeling and changes in gene expression [1]. However, selection and genetic analysis of the triticale gene pool showed that the genetic potential of rye adaptability is insufficiently realized. At the molecular level, it was found that 9% of genes in octoploid triticale and up to 30% of genes in hexaploid triticale become silent [2], which may be one of the reasons for the incomplete realization of hybrid genetic potential.

The synthesis of rye-wheat amphidiploids, which have wheat (*Triticum* L.) as the pollinator and rye (*Secale* L.) as the maternal form, can offer better setting for enhancing rye gene expression and for the creation of hybrids valuable for breeding [3]. Crosses like these are normally difficult to achieve due to incompatibility, and so rye-wheat amphiploids are not yet studied. Overcoming this barrier is usually associated with severe rearrangements in the genomes of parental forms, which is the most vivid manifestation of the “genomic shock” that occurs when parents' genomes are combined in a hybrid cell [4] and is accompanied by various chromosomal abnormalities, including those affecting the centromere structure. It has been previously determined that using an intermediary species triticale as a source of wheat genomes in crosses with rye proved to be effective for overcoming the barrier of unidirectional progamic incompatibility of parent species [3]. As a result, these rye-wheat amphidiploid hybrids were created by crossing tetraploid rye (^S/^RRRR, 2n = 4x = 28) with hexaploid triticale (^T/^RRAABB, 2n = 6x = 42) and were named secalotriticum (×Secalotriticum, syn. ×Secalotriticum =* Secale* L. ×* Triticum* L., ^S/^RRAABB, 2n = 6x = 42). The presence of rye maternal cytoplasm in hybrids was proved by analyzing restriction fragments of species-specific mitochondrial DNA18S/5S-repeated sequences and the* ndhH*-region of chloroplast DNA, which showed a pattern characteristic of chloroplast DNA and rye mitochondrial DNA [3]. The created secalotriticum hybrids are characterized by a wider variability range of morphological and economically valuable traits compared to the original triticale forms [3]. Seсalotritiсum creates more advantageous conditions for enhancing the expression of rye genetic systems and the manifestation of its valuable adaptive traits.

The process of the hybrid genome formation and its subsequent stabilization are directly related to the normalization of meiosis and proper segregation of chromosomes. Incompatibility of centromeres of different species seems to be the main reason of the chromosome elimination of one of the parental genomes in hybrids [5, 6]. The pivotal role in the proper chromosome segregation during meiosis and mitosis lies with centromeres, their identity being defined by the presence of the centromere-specific variant of histone H3 known in plants as centromere-specific histone H3 variant CENH3 [7, 8]. This is due to the fact that at the molecular level the most specialized and universal characteristic of the active centromere is the presence of CENH3 instead of the canonical histone H3 in the nucleosomes of centromeric chromatin. As it has been shown in some mammalian species and* Drosophila*, in case of centromeric histone loss, there is no kinetochore formation and no correct chromosome segregation during cell division for some reason [9]. Unlike canonical histone Н3, which has a conserved structure, CENH3 normally shows considerable variability across species [5, 10, 11]. Different domains of this molecule are diverging differently. An extended N-terminal tail (NTT) domain and loop 1 of the histone fold domain (HFD) putatively interact with centromeric DNA [12] and show signatures of positive selection in some animal and plant species [13], while the part of the HFD that lies outside loop 1 is generally conserved [14, 15].

Because of a special role CENH3 has in the formation and function of centromeres, we explore effects of remote hybridization using an unusual combination of parental forms on the structure of this protein and perform a cytogenetic analysis of the karyotypes.

## 2. Materials and Methods

Two secalotriticum combinations (×*Secalotriticum* Rozenst., ^S/^RRAABB, 2n = 6x = 42) Verasen' × Mikhas' (STr VМ) and Verasen' × Dubrava (STr VD) were developed by hybridizing the tetraploid rye cultivar Verasen' (^*S*/^RRRR, 2n=4x=28) with the hexaploid triticale cultivars Mikhas' and Dubrava (^*T*/^AABBRR, 2n=6x=42). Triticale acted as an intermediary species, a source of wheat genomes and an inhibitor of rye S-RNases, which allowed overcoming the rye progamic incompatibility with wheat. Stabilization of karyotypes was facilitated by a single backcross of rye-triticale pentaploid F1 hybrids (S/RRABR, 5x=35) on triticale followed by self-pollination within 15 generations and constant selection for cytological stability and phenotypic homogeneity.

Karyotyping of secalotriticum, identification of the original species chromosomes was carried out by means of root apical meristem squashed preparations and differential staining using a Giemsa technique (C-banding) [16].

Molecular analysis was conducted on plants of parental forms and hybrids grown in a greenhouse. The total RNA was isolated from leaves of 10-12-day-old seedlings from 4-5 plants of parental forms. RNA of hybrid forms was isolated from each plant separately. Isolation of RNA was performed using the TRI Reagent (MRC, Ink., USA) [17]. To avoid contamination with genomic DNA, total RNA was treated with RQ-RNase_Free DNase (Promega, Madison, USA). To synthesize cDNA, a RevertAidTM H Minus First Strand cDNA Synthesis Kit (Thermo Scientific, USA) was applied. The resulting cDNA was used as a template in a series of PCR reactions with primers synthesized specifically to amplify the CENH3 N-terminal tail (NTT). The primer sequences are 5′ – ATGGCCCGCACCAAGC (F) and 5′ – GAAACTCGACCGACTTCTG (R). The product size was 268 bp. PCR products were cloned into the pTZ57R/T plasmid (InsTAclone PCR cloning kit, Thermo Scientific, США) and analyzed by Sanger sequencing using BigDye Terminator v3.1 Cycle Sequencing Kit (Applied Biosystems, USA). The reaction products were separated on the 3500 Genetic Analyzer (Applied Biosystems, USA). The sequences of individual clones obtained for each sample were analyzed using the UniPro Ugene software (http://ugene.net) and FASTA software package [18]. The search for the identity of the nucleotide sequences was carried out using the BLAST algorithm in the NCBI database (http://blast.ncbi.nlm.nih.gov/Blast.cgi). Amino acids alignments were performed online using Clustal Omega (http://www.ebi.ac.uk/Tools/msa/clustalo) and used for downstream analysis and visualization (http://www.jalview.org). Graphic images were prepared using the Jalview program (http://www.jalview.org/).

## 3. Results and Discussion

**Figure 1:**
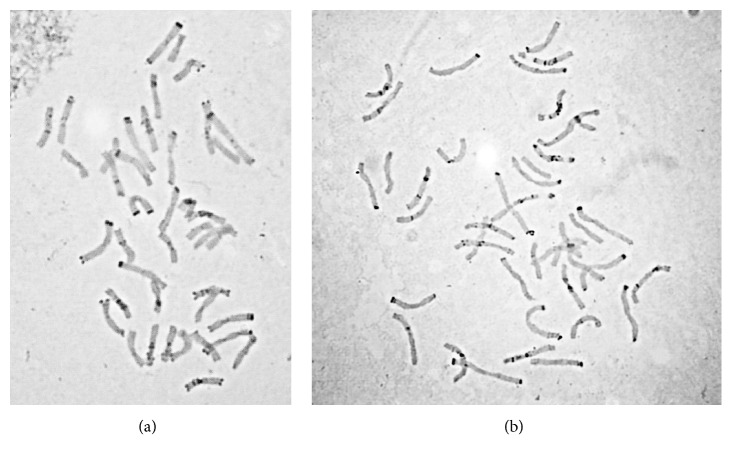
The STr VM (a) and STr VD (b) karyotypes of hybrid secalotriticum following C-banding appear with the whole sets of** R**,** A**, and** B **genomes.

The STr VM (Verasen' × Mikhas') and STr VD (Verasen' × Dubrava) (S/RRAABB, 2n=6x=42) secalotriticum karyotypes were analyzed by differential staining (C-banding) [16]. This method reveals the pattern of heterochromatic regions localization, which is specific for each chromosome of rye and wheat, thereby allowing identification of each chromosome in a hybrid karyotype. Figures 1(a) and 1(b) show that the karyotypes of the STr VM and STr VD hybrids are stable balanced hexaploids not containing minichromosomes with deleted arms, in full or in part. The hybrids and the original rye and wheat forms have an identical C-banding pattern.

One of the genomic shock manifestations, which arise from the combination of two parental genomes in a hybrid cell in case of remote hybridization, is various chromosomal aberrations. Deletions and translocations of individual chromosomal regions and chromosome arms are among the most common changes and have been found in the cytogenetic analysis of both wheat-rye substitution and addition lines [19, 20] and in triticale and offspring from crosses of triticale × wheat [21, 22]. The disorders herewith have been documented in the chromosomes of both parental forms. Stabilization of secalotriticum karyotypes was assisted by a single backcross of rye-triticale pentaploid F1 hybrids (S/RRABR, 5x=35) on triticale with subsequent self-pollination during 15 generations and constant selection for cytological stability and phenotypic homogeneity. Diploid RR-rye genome in rye-triticale F1 hybrids is a factor of meiosis stabilization and provides functionality of gametes with different chromosome composition. The formation of the secalotriticum genome takes place in F1BC1 on the basis of partially unreduced 21-chromosome gametes of F1 pentaploids with balanced chromosome sets of the original species haplogenomes (7(R), 7(A), 7(B)). Equation division (division into chromatids) of asynaptic univalents in anaphase I (AI) is the main source of chromosomal abnormalities during the second meiosis division (MII): individual chromatids are not included in the metaphase plate in MII; in anaphase II they often lag behind other chromosomes in division, segregate randomly, undergo fragmentation, and form micronuclei in the microspore tetrads, which leads to the elimination of genetic material and aneuploidy and the genomic instability [3, 23, 24]. Stability of the secalotriticum genome and its genetic diversity are determined by the cytoplasm type and some factors, apparently related to the structural and functional state of centromeres in its genome. Analyzing meiosis in hybrids, we observed the formation of (pseudo)univalents mainly as a result of desynapsis [23, 24]. Desynapsis here means the early decomposition of bivalents into univalents of desynaptic origin (pseudounivalents) that occur in the late prophase (prometaphase I) of meiosis. Unlike the asynaptic univalents characteristic of triticale, the pseudounivalents maintain their unipolar centromere orientation in the reductional (I) division of meiosis. The equational (II) division of meiosis was characterized by regular polar segregation and a low level of chromosome elimination [23, 24].

The secalotriticum forms created underwent rapid stabilization in meiosis during selection for productivity. Stabilization was due to the narrowing of the spectrum and a rapid decrease in the frequency of the chromosome abnormalities in the second meiotic division (by 15-50%) in F1-F3, including the level of chromosome elimination (from 30% to 6% tetrads with micronuclei). In F3-5 and subsequent secalotriticum generations, the normalization of the first division of meiosis was more pronounced: the average level of anomalies in MI metaphase decreased from 57.3% in F3 to 16.9% in F5 and also from 25-30% up to 8-10% in AI-AII anaphases and reached less than 5% at the tetrad stage [3, 23, 24]. The fact that the chromosomal changes mentioned above tend to occur quickly and most intensively in the first generations, up to the fifth, inclusive, and especially in F1 before the chromosome doubling, is probably a general trend in remote hybridization of cereals [3, 21, 23, 24]. Thus, continuous cycles of crosses in the secalotriticum production played a significant role in stabilizing the hybrid karyotype and replacing possible lost parts of the parental genomes in the first generations after hybridization.

Differences in the CENH3 structure between the parents allow estimating the expression level of the parental forms of the protein in a new genomic environment that arises in a hybrid cell under remote hybridization. The first work on studying possible connection between differences in the CENH3 structure in parental forms and the processes of parental genomes chromosome segregation during hybrid cell division was carried out on hybrids obtained from the crossing of cultured barley* H. vulgare* and its closest wild relative* H. bulbosum* [5]. The CENH3 molecules were not incorporated in the centromeres of* H. bulbosum* chromosomes, which were herewith inactivated and eliminated from hybrid embryos. Perhaps the absence of CENH3 in the centromeres of* H. bulbosum* chromosomes is caused by perceptible differences in the structure of this protein between barley species, especially in the structure of the N-terminal tail (NTT) [5]. Unlike barley species, DNA sequences of *α* and *β* CENH3 forms of various rye and wheat species have a very high identity (95-99%) [25], which significantly complicates the search for interspecific differences and, accordingly, the identification of the nature of their inheritance in hybrid genomes.

**Figure 2:**
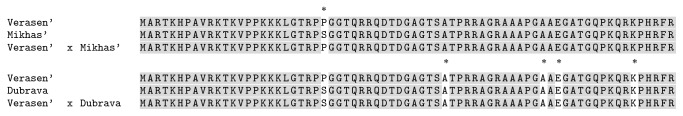
The amino acid consensus sequences of *α*CENH3 NTT domain derived from parental forms of rye (Verasen'), triticale (Mikhas', Dubrava), and secalotriticum amphidiploids (STr VM and STr VD). The asterisks indicate the positions of nonsynonymous amino acid substitutions. Amino acid polymorphic positions are color-coded according to their frequency: white, if below 50%; light-gray, if above 50%. In the positions 41, 54, 56, and 66, the percentage of substitutions does not exceed 50%, although it has high values for Dubrava and the hybrid; therefore the amino acids with the highest percentage are shown in these positions. The exact quantification of amino acid substitutions in the polymorphic positions is provided in Figure 3.

**Figure 3:**
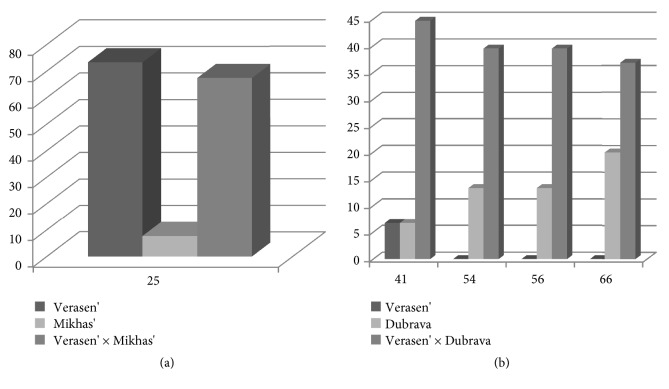
Quantitative determination of amino acid polymorphism in the NTT domain of *α*CENH3, leading to nonsynonymous amino acid substitutions. On the* x*-axis, the position in the amino acid sequence; on the* y*-axis, the percentage of clones having substitutions in polymorphic positions to the total number of clones sequenced. (a) Parental forms: Verasen' and Mikhas'; hybrids: Verasen' × Mikhas'. (b) Parental forms: Verasen' and Dubrava; hybrids: Verasen' × Dubrava.** Note:** fifteen cDNA clones were sequenced for each parental plant and the deduced amino acid sequences were obtained. About 40-45 clones were sequenced for each hybrid plant.

Consensus amino acid sequences derived from sequenced cDNA clones obtained from the *α*CENH3 NTT of Verasen' and Mikhas' parental forms differ only in one nonsynonymous substitution of amino acid serine to proline at position 25 (Figure 2). It was found in 73.3% of the analyzed clones in the Verasen' variety (Figure 3(a)), but only in 7.7% of the paternal variety Mikhas' clones. The secalotriticum cultivar Verasen' × Mikhas' inherits a high content of proline from the maternal form Verasen' (67.4%, Figure 3(a)). However, a different cross does not support this tendency: Verasen' × Dubrava has as low percentage of substitutions (2.6%) as the donor triticale cultivar Dubrava (6.7%). On the other hand, Dubrava typically has more polymorphism than the donor Verasen' at several nucleotide positions, leading to the following amino acid substitutions: alanine to valine, alanine to glycine, glutamine acid to glutamine, and lysine to asparagine (Figures 2 and 3(b)). Interestingly, although the percentage of substitutions does not exceed 50% (of the total number of clones sequenced), this value is much higher in the secalotriticum cultivar Verasen' × Dubrava than in Dubrava, something like a case of heterosis (Figure 3(b)). Thus, the results obtained indicate that both parental genomes participate in the production of the CENH3 protein in the secalotriticum hybrids. At the same time, comparison of the nucleotide and amino acid sequences of the most variable N-terminal tail of the CENH3 molecule in parental forms and hybrids does not reveal a definite tendency in inheritance.

Published data on the regulation of the expression of different genes and other classes of DNA sequences in hybrid genomes obtained by crossing different species or contrast populations of one species indicate a complex and heterogeneous inheritance pattern. The phenomenon of nucleolus dominance, characteristic for the expression of ribosomal RNA genes in triticale, as an example of genomic dominance, consists in the repression of rye rRNA genes by means of cytosine methylation [26]. However, this type of inheritance, namely, the genomic dominance of one of the parents, is not typical of most other examples. The results of gene expression study in hybrids between different ecotypes of Arabidopsis are contradictory. In one case, a general maternal dominance has been identified [27], while in the other it has been found that the maternal and paternal genomes are transcriptionally equivalent [28]. Also, it has been demonstrated that various hybrid combinations show significant variations in activation of parental alleles [29]. In some hybrids between Arabidopsis ecotypes heterosis for biomass and seed yield has been documented [30]. In these hybrids, 95% of active genes show an intermediate expression level. Among most other nonadditively expressed genes, the expression shift was towards the maternal parent [30]. When studying transcription and epigenetic adaptation of maize chromosomes in supplemented oat-maize lines containing the complete oat genome and individual maize chromosomes, most maize genes showed transcription specific for maize, but repression of gene activity was a more common trend than activation [31].

The level of parental gene expression in allopolyploid hybrids obtained by crossing wheat and rye species is of significant interest for us. We do not know such studies conducted on secalotriticum; however, an extensive analysis of rye gene expression using cDNA isolated from various tissues of hybrid plants has been carried out on allohexaploid triticale obtained from the* T. turgidum* ×* S. cereale* cross [1]. The classes of absent or silent rye genes have been identified. A comparison between diploid rye and hexaploid triticale revealed 112 rye cDNA contigs (~ 0.5% of the total amount), which were not determined by expression analysis in any of the triticale tissues, though their expression was relatively high in rye tissues. The average DNA sequence identity between rye genes not detected in triticale and their most similar contigs in the A and B genomes in* T. aestivum* was only 81%. This degree of identity was significantly lower than the global average of 91% identity between the set of 200 randomly selected rye genes and their best matches in the A and B genomes of* T. aestivum*. Thus, rye genes with a low similarity to their homeologs in* Triticum* genomes have a higher probability of being repressed or absent due to deletions in the genomes of allopolyploids. This conclusion is in good agreement with our results. High identity of rye and wheat CENH3 sequences (99%) enables a high level of expression of both parental forms in secalotriticum hybrid genomes.

## Data Availability

The data used to support the findings of this study are available from the corresponding author upon request.
